# Loneliness: Its Correlates and Association with Health Behaviours and Outcomes in Nine Countries of the Former Soviet Union

**DOI:** 10.1371/journal.pone.0067978

**Published:** 2013-07-04

**Authors:** Andrew Stickley, Ai Koyanagi, Bayard Roberts, Erica Richardson, Pamela Abbott, Sergei Tumanov, Martin McKee

**Affiliations:** 1 European Centre on Health of Societies in Transition, London School of Hygiene and Tropical Medicine, London, United Kingdom; 2 Stockholm Centre on Health of Societies in Transition (SCOHOST), Södertörn University, Huddinge, Sweden; 3 School of Social Sciences, University of Aberdeen, Aberdeen, United Kingdom; 4 Centre for Sociological Studies, Lomonosov Moscow State University, Moscow, Russia; California Pacific Medicial Center Research Institute, United States of America

## Abstract

**Background:**

Research suggests that the prevalence of loneliness varies between countries and that feeling lonely may be associated with poorer health behaviours and outcomes. The aim of the current study was to examine the factors associated with loneliness, and the relationship between feeling lonely and health behaviours and outcomes in the countries of the former Soviet Union (FSU) – a region where loneliness has been little studied to date.

**Methods:**

Using data from 18,000 respondents collected during a cross-sectional survey undertaken in nine FSU countries – Armenia, Azerbaijan, Belarus, Georgia, Kazakhstan, Kyrgyzstan, Moldova, Russia and Ukraine – in 2010/11, country-wise logistic regression analysis was conducted to determine: the factors associated with feeling lonely; the association between feeling lonely and alcohol consumption, hazardous drinking and smoking; and whether feeling lonely was linked to poorer health (i.e. poor self-rated health and psychological distress).

**Results:**

The prevalence of loneliness varied widely among the countries. Being divorced/widowed and low social support were associated with loneliness in all of the countries, while other factors (e.g. living alone, low locus of control) were linked to loneliness in some of the countries. Feeling lonely was connected with hazardous drinking in Armenia, Kyrgyzstan and Russia but with smoking only in Kyrgyzstan. Loneliness was associated with psychological distress in all of the countries and poor self-rated health in every country except Kazakhstan and Moldova.

**Conclusions:**

Loneliness is associated with worse health behaviours and poorer health in the countries of the FSU. More individual country-level research is now needed to formulate effective interventions to mitigate the negative effects of loneliness on population well-being in the FSU.

## Introduction

Loneliness has been defined as the subjective perception of either quantitative or qualitative deficiencies in an individual’s network of social relations [1]. Over the last 30 years, research on this phenomenon has grown considerably. This has been underpinned by the realisation that loneliness is widespread in contemporary society [2], can affect all age groups [3], [4], and can be an extremely painful and distressing experience [5] with serious negative health consequences. It has been linked, for example, to hazardous health behaviours such as smoking, alcohol consumption and problems [2], [6], [7], [8], increased use of health services [9], [10], and worse self-rated, physical, and mental health [11], [12], [13], [14], [15]. Research from both Europe and the United States has also linked loneliness to higher mortality [16], [17], with one recent study showing that lonely individuals over age 50 had a 1.56 to 1.83 times increased risk for all-cause mortality compared to their non-lonely counterparts [18].

Despite the seeming importance of loneliness as a determinant of health outcomes, this relationship remains little studied outside the confines of Western Europe and North America. In an attempt to partly fill this research gap, the current study will examine the relationship between loneliness and health in the countries of the former Soviet Union (FSU). The countries in this region provide an ideal environment in which to study loneliness. The collapse of the Soviet Union was followed by social, economic and political changes that had a profound impact on all aspects of daily life. Economic liberalisation and the withdrawal of the social safety nets formerly provided by the Soviet state were accompanied by growing unemployment, increasing inequality and high levels of poverty in many of these countries [19]. The sense of social chaos unleashed by these changes was further exacerbated by large-scale population movements either in search of work or as a result of the formation of nation states and the inter-ethnic tensions and/or wars that accompanied this process.

Although the situation improved after the end of the 1990s with strong economic growth recorded in the FSU countries in the following years, these improvements were not distributed evenly between these countries or different segments of the population within the countries [20]. Instead, there have been many people in this region who have been economic losers in the transition process [21], with life for some of them becoming little more than a daily struggle for survival [22]. In these conditions, distrust of the state and its institutions – a remnant of the Soviet period – has made it increasingly preferable to rely on kin and friendship networks for support [23]. However, there is some evidence that the socioeconomic changes that have made network connections so important may have also acted to undermine them. A sharp growth in rates of divorce [20], [24], high levels of male mortality [25], inter-country migration, and a decline in close relationships with neighbours [22] may have all fed through to increased social isolation and loneliness. Evidence for this proposition comes from several recent studies which suggest that rates of loneliness in some former Soviet countries may be the highest in Europe [26], [27].

In turn, the effects of a breakdown of social relations and contacts may be especially severe in terms of well-being in the FSU countries. This is because in conditions of economic difficulty networks are used to obtain essential goods and services such as medical care [28]. Indeed, they have even been linked to the ability of some people to survive in this region [29]. In such circumstances, it is possible that loneliness might not only have a direct impact on well-being via several mechanisms that have been invoked to link loneliness and low physiological resilience across time (e.g. poorer health behaviours and higher levels of perceived stress) [30], but also, that its effects on health might be being exacerbated by a variety of other problems that arise from an absence of social connections in these extremely difficult conditions.

Given the paucity of research to date about either loneliness or its association with health in the countries of the FSU, the aim of the current study was twofold: (1) to determine the prevalence of, and factors associated with feeling lonely in nine FSU countries; and (2) to examine the relationship between feeling lonely and health behaviours (alcohol consumption, hazardous drinking and smoking) and outcomes (self-rated health and psychological distress) in these countries. Determining the factors associated with feeling lonely and its relation with health outcomes across a number of countries that differ not only culturally, ethnically, religiously, and economically, but also in terms of the way their populations have reacted to the changes that have occurred in the post-Soviet period [31], is an essential task when it comes to gaining a better understanding of the relation between loneliness and health and whether it varies between countries.

## Methods

### Ethics Statement

Ethical approval for the research was obtained from the ethics committee of the London School of Hygiene and Tropical Medicine. The research was carried out in accordance with the ethical standards laid down in the 1964 Declaration of Helsinki. Written informed consent was obtained from all participants before their inclusion in the research.

### Participants

The data in this paper were drawn from the Health in Times of Transition (HITT) study. Nationally representative cross-sectional surveys were conducted with adult respondents (aged 18 years and over) in Armenia, Azerbaijan, Belarus, Georgia, Kazakhstan, Kyrgyzstan, Moldova, Russia, and Ukraine. Multi-stage random sampling with stratification by region and rural/urban settlement type was used. Within each primary sampling unit (about 100–200 per country), households were selected by random route procedures. Within each of the selected households, one person was chosen (based on the nearest birthday). If after three visits (on different days and times) there was no one at home, the next household on the route was selected. Some pre-specified quota control was used in Belarus, Kazakhstan, Moldova and Ukraine (a combination of region, area, gender, age and/or education level).

The surveys were conducted between March and May 2010 (with the data collection in Kyrgyzstan delayed until early 2011 due to political violence). Face-to-face interviews were conducted by trained fieldworkers in the respondents' homes. Response rates varied from 47% in Kazakhstan to 83% in Moldova. There were 1800 respondents in each country, except in Russia (n = 3000) and Ukraine (n = 2000) where more respondents were recruited to reflect their larger and more regionally diverse populations, and in Georgia (n = 2200) where a booster survey of 400 additional interviews was undertaken in November 2010 to ensure a more representative sample. All persons gave their informed consent prior to their inclusion in the study.

The draft questionnaire was forward and back translated into each of the languages in which it was administered, and then piloted before being finalised. Except in Russia and Belarus (where all interviews were conducted in Russian), respondents were given the choice of answering in Russian or a national language.

### Measures

Loneliness was assessed by a single item question ‘How often do you feel lonely?’ with four response options: ‘Often’, ‘Sometimes’, ‘Rarely’ and ‘Never’. Following a recent study which has shown that often feeling lonely carries an increased risk for worse health outcomes [16], we dichotomised this variable into those who replied ‘Often’ (coded ‘1’) and those with other response options (coded ‘0’).

Using previous research as a guide, a number of different socio-demographic and other variables were examined as potential correlates of loneliness. Age was divided into five categories: 18–29, 30–39, 40–49, 50–59 and 60 years and above. Marital status was divided into three categories: ‘Married/Cohabiting’, ‘Single’ (never married) and ‘Divorced/Widowed’. Educational level was grouped into three categories: ‘High’ (incomplete high or high education), ‘Middle’ (completed secondary education) and ‘Low’ (incomplete secondary education or below). Residential location was categorised as either ‘Urban’ or ‘Rural’. Household size was determined by asking respondents the question, ‘How many people constantly live in your household?’ with the variable being dichotomised into one person (coded ‘1’) or two and above (coded ‘0’). Difficulty in undertaking physical activity (‘Physical activity difficulty’) was assessed by asking the respondents how easily they could ‘go up two or three flights of stairs or go uphill without getting out of breath’ with the response categories being ‘Very easily’, ‘Fairly easily’, with ‘Some difficulties’ and with ‘Major difficulties’. This variable was dichotomised as ‘Major’ (i.e. Major difficulties) (coded ‘1’) or ‘No major difficulties’ (coded ‘0’). Information on psychological perceptions of control (i.e. locus of control) was obtained by asking the respondents to what extent they agree with the following statement: ‘I feel that what happens in my life is often determined by factors beyond my control’; with the response options ‘Disagree’, ‘Rather disagree’ (categorised together as signifying a ‘High’ degree of control), ‘Quite agree’ (categorised as ‘Middle’) and ‘Agree’ (categorised as ‘Low’). Principal component analysis was used to generate a wealth index based on the ownership of ten assets. This was subsequently divided into the wealth tertiles ‘High’, ‘Middle’ and ‘Low’ in terms of the respondents’ asset ownership. Respondents’ level of social support was determined by asking them three questions: ‘Is there anyone who you can really count on to listen to you when you need to talk?’, ‘Is there anyone who you can really count on to help you out in a crisis/in your most difficult moments?’ and ‘Is there anyone who can comfort you when you are very upset?’. The response options were ‘Yes’ and ‘No’. Those respondents who answered ‘Yes’ to all three questions were categorised as having a ‘High’ level of social support, those answering ‘Yes’ to two questions were categorised as having a moderate (‘Middle’) level of social support, while those who answered ‘Yes’ to only one or none of the questions were categorised as having a ‘Low’ level of social support. Finally, the effect of bereavement was assessed by asking respondents if they had experienced the death of a close relative in recent months.

#### Health behaviours and outcomes

Alcohol consumption, hazardous drinking and smoking were the health behaviours examined. Information on alcohol consumption was obtained by asking respondents, ‘How often do you consume alcoholic drinks of any type’ with eight main response categories ranging from ‘Every day’ to ‘Never’. All subjects who did not use the response option ‘Never’ were categorised as drinkers. Hazardous drinking was examined using two measures – heavy episodic drinking and problem drinking. As regards the former, we followed the definition of a previous study that has examined this phenomenon in the FSU (i.e. drinking at least one of: ≥2 litres of beer, ≥750 grams of wine, or ≥200 grams of strong spirits on one occasion) [32]. Problem drinking was assessed using the CAGE questionnaire [33]. This consists of four questions relating to the potentially negative effects of drinking (‘Have you ever felt you should cut down on your drinking?’, ‘Have people annoyed you by criticising your drinking?’, ‘Have you ever felt bad or guilty about your drinking?’, ‘Have you ever had a drink first thing in the morning to steady your nerves or to get rid of a hangover?’) with two or more ‘Yes’ answers indicating problem drinking. Previous studies have validated this instrument for determining alcoholism [34] and it is now a commonly used measure for detecting alcohol problems (Cronbach’s α = 0.75). Respondents were also asked ‘Do you smoke at least one cigarette (papirossi, pipe, cigar) per day?’. Those who answered in the affirmative were categorised as smokers.

Self-rated health scores were obtained by asking respondents the question, ‘In general would you say your health is…’ with the response options, ‘Very good’, ‘Good’, ‘Fair’, ‘Poor’ and ‘Very poor’. These were categorised as ‘Poor’ self-rated health (comprising the ‘Poor’ and ‘Very poor’ categories (coded ‘1’)) and ‘Good/Fair’ self-rated health (comprising the remaining categories (coded ‘0’)). To assess psychological distress, we used a slightly modified version of a measure that has been employed in several previous studies in the countries in this region [35], [36]. Modification was necessary because a question on loneliness was included in the original 12-item scale. The modified scale consisted of 11 items that encompassed a range of negative psychological feelings: (1) ‘Inability to concentrate’, (2) ‘Insomnia’, (3) ‘Constant feelings of strain’ (4) ‘Inability to overcome difficulties’, (5) ‘Losing confidence’, (6) ‘Shaking nervously or trembling’, (7) ‘Having frightening thoughts’, (8) ‘Experiencing exhaustion or fatigue’, (9) ‘Feeling stress’, (10) ‘Feeling an impossibility to influence things’, and (11) ‘Feeling that life is too complicated’. For each item, subjects could answer either ‘Yes’ or ‘No’ if they had experienced the symptom in recent weeks. This created a composite psychological distress score running from 0–11 (Cronbach’s α = 0.80). For the psychological distress outcome, those subjects who fell into the top quintile of scores in terms of suffering most distress (which equated to a score of 6 or above on the psychological distress scale) were scored ‘1’ while the remainder of the subjects were scored ‘0’.

### Statistical Analyses

Multivariate logistic regression was used to examine the association between loneliness and its potential correlates (sex, age, marital status, education, location, household size, physical activity difficulty, locus of control, wealth, social support, and death of a close relative) in each country. The models were mutually adjusted for all these 11 covariates. The association between loneliness and health behaviours (alcohol consumption, hazardous drinking and smoking) and outcomes (poor self-rated health and psychological distress) was also estimated by country-wise multivariate logistic regression where the models were also adjusted for the 11 variables examined as potential correlates of loneliness.

All analyses were carried out using the statistical software package Stata 12.0 (Stata Corp LP, College Station, Texas). All of the results of the regression analyses are presented in the form of odds ratios (OR) with 95% confidence intervals (CI). The level of statistical significance was set at p<0.05.

## Results

### Baseline Characteristics of the Study Sample and the Prevalence of Loneliness

**Figure 1:**
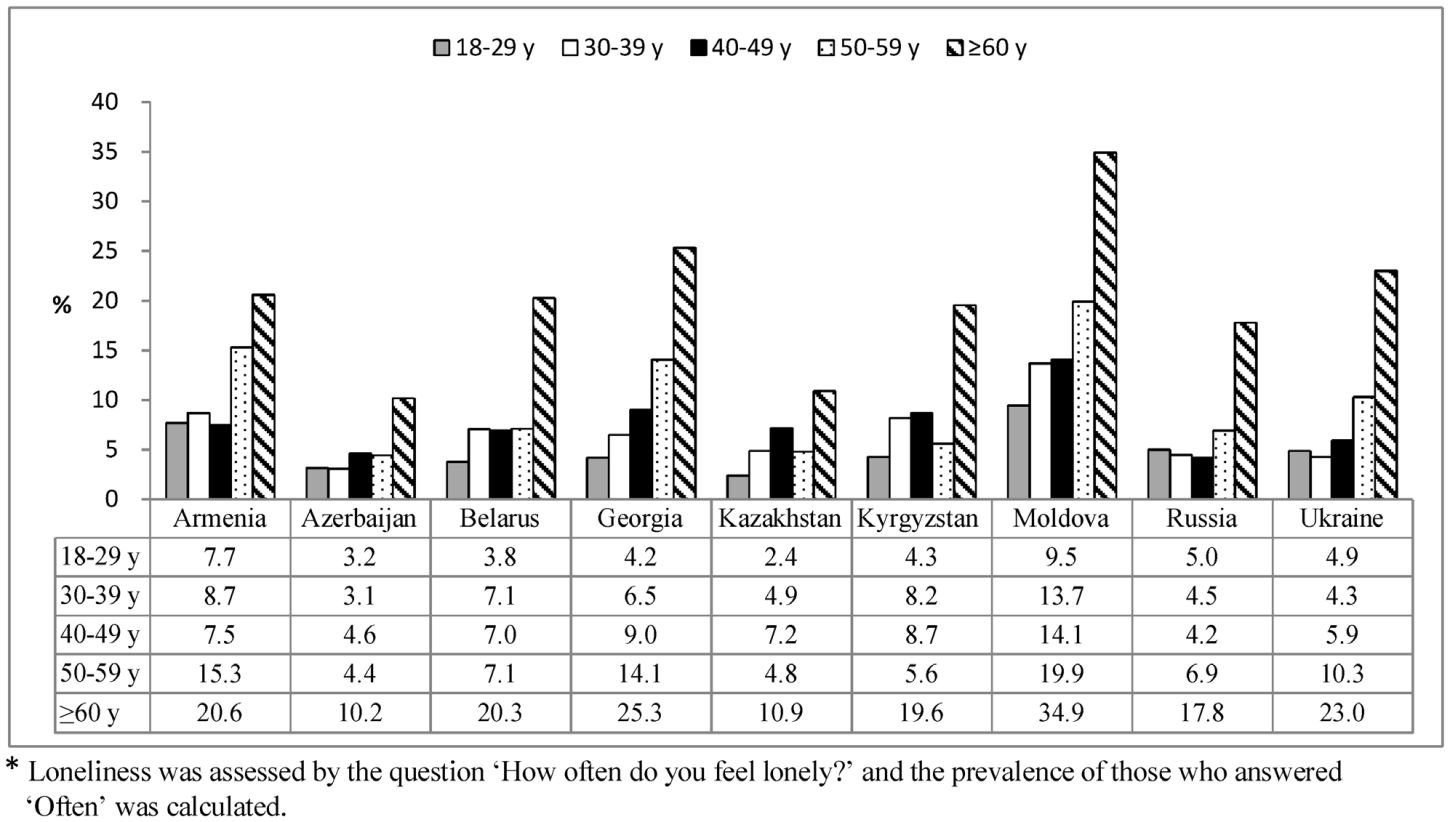
Prevalence of loneliness by country and age.

The sample characteristics are shown in Table 1. There were more women than men in every country with most respondents (55% to 69%) being married and having a middle level of education. Rural respondents predominated in only three countries – Georgia, Kyrgyzstan and Moldova. The percentage of respondents who lived alone varied widely between the countries ranging from 2.2% in Azerbaijan to 15.4% in Ukraine. As regards the prevalence of loneliness, across the countries, 4.4% (Azerbaijan) to 17.9% (Moldova) of respondents reported that they often felt lonely with four countries having a prevalence in excess of 10% - Armenia (10.7%), Ukraine (10.8%), Georgia (12.3%) and Moldova. In every country, the highest prevalence of loneliness was observed among those aged 60 years-old and above (see Figure 1). In five of the countries, the youngest age group was least likely to report feeling lonely.

**Table 1:** Characteristics of the study sample (N = 18000).

Characteristic	Categories	Armenia	Azerbaijan	Belarus	Georgia	Kazakhstan	Kyrgyzstan	Moldova	Russia	Ukraine
Sex	Male	45.7	47.0	43.6	36.4	47.4	48.3	44.3	40.4	42.2
Age (years)	18–29	30.6	34.7	28.2	19.7	30.5	34.3	28.2	24.4	25.9
	30–39	20.4	18.7	19.0	19.2	23.0	23.1	15.9	17.3	15.5
	40–49	22.1	22.8	17.6	19.7	17.7	18.6	17.5	17.6	16.3
	50–59	12.6	14.0	14.3	17.5	14.0	13.0	18.7	17.3	14.7
	≥60	14.2	9.8	20.9	24.0	14.8	11.1	19.7	23.4	27.7
Marital status	Married/cohabiting	65.4	65.4	55.5	63.1	62.7	68.9	62.4	59.7	57.2
	Single	26.2	27.2	23.8	17.9	21.9	18.9	17.5	17.2	17.8
	Divorced or widowed	8.3	7.4	20.7	19.0	15.4	12.2	20.1	23.2	25.0
Education†	High	22.0	24.8	25.7	38.4	28.7	23.2	23.0	26.2	33.6
	Middle	69.2	67.7	66.6	53.7	59.9	53.5	49.1	61.0	54.9
	Low	8.8	7.5	7.7	8.0	11.4	23.3	27.9	12.9	11.5
Location	Rural	22.6	43.6	26.5	52.2	44.4	54.4	61.8	27.4	30.2
Household size	Living alone	2.3	2.2	14.4	10.3	5.7	4.1	12.8	13.6	15.4
Physical activity difficulty*	Major	10.8	17.0	7.5	20.5	11.8	11.1	12.6	10.3	13.5
Locus of control§	High	33.4	35.9	43.8	27.7	44.5	33.5	38.5	43.9	39.0
	Middle	38.8	34.6	36.0	45.2	31.0	32.8	34.9	32.4	36.4
	Low	27.7	29.5	20.1	27.2	24.5	33.7	26.7	23.7	24.6
Wealth‡	High	28.9	14.4	50.9	23.8	29.6	15.1	28.7	45.1	37.2
	Middle	36.4	59.2	29.6	36.6	36.6	38.5	25.6	30.1	29.2
	Low	34.7	26.4	19.6	39.6	33.8	46.4	45.7	24.8	33.7
Social support¶	High	71.6	85.4	89.6	92.8	89.0	88.2	83.6	90.8	88.7
	Middle	13.4	6.9	5.7	3.8	6.7	6.8	5.3	4.3	5.5
	Low	15.0	7.6	4.7	3.4	4.3	5.0	11.1	4.9	5.8
Death of close relative#	Yes	19.7	16.6	12.3	30.6	15.4	13.1	19.1	14.0	15.6
Loneliness	Often	10.7	4.4	8.9	12.3	5.4	7.9	17.9	8.1	10.8

Data are %.

† Education was classified as: low (less than complete secondary education), middle (completed secondary education), high (incomplete or complete higher education).

* Physical activity difficulty was assessed by the question ‘How easily can you go up 2 or 3 flights of stairs or go uphill without getting out of breath?’

§ Answers to the question ‘I feel that what happens in life is often determined by factors beyond my control’ were classified as: disagree/rather disagree (high), quite agree (middle), agree (low).

‡ Principal component analysis was used to generate the wealth index based on the possession of 10 household assets.

¶ Level of social support was based on a composite score (refer to text for details).

# Death of a close relative was assessed by the question ‘During the last months, have you experienced the death of a close relative?’

### Correlates of Loneliness

In the fully adjusted multivariate logistic regression analysis (Table 2), women were significantly more likely than men to report feeling lonely in four of the nine countries – Belarus, Georgia, Kazakhstan and Ukraine, with odds ratios ranging from 1.52 (Georgia) to 2.28 (Belarus) in these countries. There was almost no independent age effect, except in Belarus where those aged 60 years-old and above were 2.4 times more likely to report loneliness when compared with their 18–29 year-old counterparts. Being single (never married) more than doubled the odds of reporting loneliness in Georgia (OR: 2.17; CI: 1.29–3.63), and more than tripled them in Ukraine (OR: 3.11; CI: 1.48–6.52). In contrast, being divorced or widowed was associated with an increased risk of reporting loneliness in every country with odds ratios running from 2.43 (Ukraine) to 5.45 (Kyrgyzstan). Neither educational level nor residential location was associated with loneliness in any of the countries. In seven of the nine countries, living alone was significantly associated with loneliness, with odds ratios of over 5 recorded in Armenia, Azerbaijan, Georgia and Kyrgyzstan. Experiencing major difficulties in terms of physical activity and having a low locus of control were both linked to an increased risk of loneliness in the majority of countries, with both variables having a significant effect in Azerbaijan, Georgia, Moldova and Russia. Wealth had a more limited effect, with low levels of wealth being associated with loneliness in only three countries: Armenia, Moldova and Russia, although in Russia, even having a ‘Middle’ level of wealth was associated with a significant increase in the risk of loneliness (OR: 1.84; CI: 1.14–2.97). Having a low level of social support was also associated with an increased risk of experiencing loneliness in every country, with respondents in Belarus having very high odds in this regard. Indeed, even having only a moderate (Middle) level of social support was associated with an increased risk of experiencing loneliness in Belarus, Georgia, Russia and Ukraine compared to those with high support. The recent death of a close relative was associated with a significant increase in the odds of feeling lonely in five countries – Belarus, Georgia, Kazakhstan, Russia and Ukraine.

**Table 2:** Predictors of loneliness estimated by multivariate logistic regression.

	Armenia	Azerbaijan	Belarus	Georgia	Kazakhstan	Kyrgyzstan	Moldova	Russia	Ukraine
N	1605	1651	1677	1998	1695	1723	1667	2553	1771
Characteristics	Adj. OR (95%CI)	Adj. OR (95%CI)	Adj. OR (95%CI)	Adj. OR (95%CI)	Adj. OR (95%CI)	Adj. OR (95%CI)	Adj. OR (95%CI)	Adj. OR (95%CI)	Adj. OR (95%CI)
**Sex**									
Female	1.14 (0.79–1.65)	1.67 (0.91–3.07)	**2.28 (1.45–3.59)^c^**	**1.52 (1.03–2.24)** a	**1.72 (1.03–2.87)** a	0.97 (0.63–1.50)	1.32 (0.96–1.80)	1.40 (0.95–2.06)	**1.90 (1.23–2.95)^b^**
**Age** (years)									
30–39	1.20 (0.65–2.21)	0.85 (0.33–2.20)	2.04 (0.95–4.37)	1.42 (0.71–2.84)	1.79 (0.76–4.20)	1.75 (0.92–3.32)	1.07 (0.61–1.90)	0.78 (0.39–1.55)	0.93 (0.37–2.32)
40–49	0.94 (0.49–1.81)	0.88 (0.36–2.15)	1.55 (0.66–3.61)	1.72 (0.88–3.36)	2.13 (0.85–5.32)	1.20 (0.60–2.40)	0.78 (0.43–1.40)	0.53 (0.25–1.13)	1.49 (0.64–3.46)
50–59	1.63 (0.83–3.20)	0.64 (0.22–1.83)	1.34 (0.55–3.25)	1.83 (0.93–3.61)	1.00 (0.36–2.76)	0.63 (0.27–1.49)	0.80 (0.45–1.43)	0.70 (0.35–1.40)	1.76 (0.76–4.08)
≥60	1.43 (0.72–2.86)	0.47 (0.14–1.54)	**2.38 (1.01–5.63)** a	1.10 (0.54–2.23)	1.28 (0.46–3.55)	1.14 (0.50–2.62)	0.83 (0.44–1.55)	0.78 (0.39–1.57)	1.69 (0.75–3.80)
**Marital status**									
Single	1.46 (0.85–2.53)	1.65 (0.71–3.85)	1.59 (0.78–3.24)	**2.17 (1.29–3.63)^b^**	1.85 (0.81–4.22)	1.57 (0.80–3.06)	0.81 (0.45–1.46)	1.01 (0.52–1.94)	**3.11 (1.48–6.52)^b^**
Divorced or widowed	**3.46 (2.06–5.80)^c^**	**4.92 (2.30–10.54)^c^**	**3.75 (2.21–6.36)^c^**	**4.05 (2.66–6.17)^c^**	**5.23 (2.93–9.34)^c^**	**5.45 (3.24–9.18)^c^**	**2.62 (1.72–4.00)^c^**	**2.51 (1.59–3.95)^c^**	**2.43 (1.46–4.05)^b^**
**Education** †									
Middle	1.00 (0.63–1.60)	1.34 (0.64–2.83)	1.10 (0.66–1.83)	1.22 (0.84–1.78)	1.27 (0.69–2.34)	1.52 (0.86–2.70)	1.33 (0.87–2.04)	0.97 (0.58–1.62)	1.53 (0.95–2.46)
Low	1.00 (0.49–2.02)	1.55 (0.52–4.65)	1.11 (0.51–2.38)	1.09 (0.57–2.11)	1.23 (0.54–2.82)	1.46 (0.76–2.81)	0.92 (0.57–1.50)	1.51 (0.80–2.83)	1.29 (0.69–2.43)
**Location**									
Rural	0.81 (0.52–1.24)	1.10 (0.62–1.96)	0.70 (0.44–1.12)	0.87 (0.62–1.24)	0.66 (0.40–1.10)	1.23 (0.81–1.88)	1.35 (0.96–1.91)	1.28 (0.88–1.86)	0.92 (0.61–1.38)
**Household size**									
Living alone	**5.33 (2.37–11.99)^c^**	**5.16 (1.85–14.42)^b^**	1.51 (0.85–2.68)	**5.67 (3.65–8.81)^c^**	1.42 (0.70–2.87)	**5.42 (2.80–10.47)^c^**	**3.33 (2.11–5.25)^c^**	**2.02 (1.29–3.17)^b^**	**2.93 (1.81–4.73)^c^**
**Physical activity difficulty** *									
Major	**1.84 (1.10–3.06)** a	**2.50 (1.32–4.75)^b^**	1.28 (0.69–2.35)	**2.34 (1.60–3.42)^c^**	1.31 (0.66–2.57)	1.76 (0.98–3.15)	**1.81 (1.16–2.82)^b^**	**2.21 (1.42–3.44)^c^**	**2.11 (1.32–3.35)^b^**
**Locus of control** §									
Middle	1.15 (0.75–1.78)	**3.58 (1.57–8.20)^b^**	**1.65 (1.01–2.69)** a	1.17 (0.75–1.82)	0.98 (0.52–1.82)	1.03 (0.61–1.74)	0.73 (0.50–1.06)	**2.80 (1.77–4.42)^c^**	0.84 (0.54–1.32)
Low	1.24 (0.79–1.96)	**4.15 (1.83–9.42)^b^**	**2.96 (1.80–4.88)^c^**	**1.98 (1.26–3.12)^b^**	**2.66 (1.53–4.60)^c^**	1.26 (0.77–2.06)	**1.51 (1.05–2.16)** a	**4.70 (2.98–7.42)^c^**	1.38 (0.87–2.17)
**Wealth** ‡									
Middle	0.77 (0.45–1.33)	0.84 (0.36–1.97)	0.74 (0.44–1.25)	1.30 (0.78–2.15)	1.03 (0.51–2.09)	1.00 (0.49–2.06)	1.57 (0.99–2.49)	**1.84 (1.14–2.97)** a	0.97 (0.56–1.67)
Low	**2.46 (1.51–3.99)^c^**	1.06 (0.42–2.67)	0.93 (0.50–1.72)	1.41 (0.84–2.38)	1.87 (0.95–3.71)	1.37 (0.68–2.76)	**1.66 (1.06–2.60)** a	**1.94 (1.16–3.27)** a	1.50 (0.88–2.57)
**Social support** ¶									
Middle	1.21 (0.73–2.01)	1.29 (0.52–3.20)	**2.19 (1.15–4.14)** a	**3.62 (1.94–6.74)^c^**	1.31 (0.58–2.95)	1.64 (0.81–3.32)	1.74 (0.96–3.13)	**3.87 (2.21–6.75)^c^**	**4.39 (2.49–7.74)^c^**
Low	**1.94 (1.26–3.01)^b^**	**3.76 (1.96–7.22)^c^**	**7.57 (4.15–13.83)^c^**	**3.05 (1.61–5.78)^b^**	**4.62 (2.36–9.03)^c^**	**5.15 (2.91–9.13)^c^**	**4.81 (3.23–7.18)^c^**	**4.19 (2.55–6.89)^c^**	**4.58 (2.66–7.88)^c^**
**Death of close relative** #									
Yes	1.23 (0.81–1.89)	1.49 (0.78–2.82)	**2.24 (1.37–3.66)^b^**	**1.56 (1.11–2.18)** a	**1.95 (1.10–3.45)** a	1.01 (0.57–1.76)	1.02 (0.70–1.47)	**1.75 (1.17–2.64)^b^**	**1.68 (1.08–2.60)** a

Mutually adjusted for all covariates in the model.

Loneliness was assessed by the question ‘How often do you feel lonely?’ and those who answered ‘Often’ were coded as ‘1’ and ‘Sometimes/Rarely/Never’ as ‘0’.

Reference categories were: sex (male), age (18–29), marital status (married/cohabiting), education (high), location (urban), household size (≥2), physical activity (no major difficulties), locus of control (high), wealth (high), social support (high), death of a close relative (no).

† Education was classified as: low (less than complete secondary education), middle (completed secondary education), high (incomplete or complete higher education).

* Physical activity difficulty was assessed by the question ‘How easily can you go up 2 or 3 flights of stairs or go uphill without getting out of breath?’.

§ Answers to the question ‘I feel that what happens in life is often determined by factors beyond my control’ were classified as: disagree/rather disagree (high), quite agree (middle), agree (low).

‡ Principal component analysis was used to generate the wealth index based on the possession of 10 household assets.

¶ Level of social support was based on a composite score (refer to text for details).

# Death of a close relative was assessed by the question ‘During the last months, have you experienced the death of a close relative?’.

a P<0.05, ^b^ P<0.01, ^c^ P<0.001.

### Loneliness and Health Behaviours and Outcomes

Loneliness was associated with both an increased risk of consuming alcohol (OR: 1.64; CI: 1.07–2.50) and smoking (OR: 2.29; CI: 1.36–3.86) in Kyrgyzstan (see Table 3). It was also associated with an increased risk of problem drinking in Kyrgyzstan (OR: 2.80; CI: 1.61–4.86) and Russia (OR: 1.72; CI: 1.07–2.75). Although loneliness was associated with an increased risk of heavy episodic drinking in Armenia (OR: 2.53; CI: 1.26–5.10), in Moldova, it was associated with a significantly reduced risk for this form of alcohol consumption (OR: 0.41; CI: 0.17–0.97). As regards health outcomes, loneliness was associated with poor self-rated health in seven of the nine countries with the odds of reporting poor health more than doubling in four countries (Armenia, Azerbaijan, Kyrgyzstan and Russia) and trebling in Belarus (OR: 3.02; CI: 1.80–5.06). In every country, loneliness was associated with a heightened risk of psychological distress with the odds ratios ranging from 1.88 (CI: 1.21–2.92) in Armenia to 4.38 (CI: 2.79–6.88) in Belarus.

**Table 3:** Association between loneliness, and health behaviours and outcomes estimated by multivariate logistic regression.

		Armenia	Azerbaijan	Belarus	Georgia	Kazakhstan	Kyrgyzstan	Moldova	Russia	Ukraine
		Adj. OR	Adj. OR	Adj. OR	Adj. OR	Adj. OR	Adj. OR	Adj. OR	Adj. OR	Adj. OR
Outcome	Loneliness#	(95%CI)	(95%CI)	(95%CI)	(95%CI)	(95%CI)	(95%CI)	(95%CI)	(95%CI)	(95%CI)
**Health behaviour** †										
Alcohol consumption	N	1393	1646	1674	1994	1693	1723	1661	2546	1755
	No	1.00	1.00	1.00	1.00	1.00	1.00	1.00	1.00	1.00
	Yes	0.89 (0.58–1.35)	0.61 (0.21–1.78)	0.90 (0.54–1.48)	0.80 (0.55–1.15)	1.10 (0.66–1.85)	**1.64 (1.07–2.50)** a	0.69 (0.47–1.00)	1.01 (0.69–1.47)	0.95 (0.63–1.43)
Heavy episodic drinking	N	1605	1532	1677	1998	1695	1723	1667	2553	1771
	No	1.00	1.00	1.00	1.00	1.00	1.00	1.00	1.00	1.00
	Yes	**2.53 (1.26–5.10)^b^**	2.57 (0.65–10.21)	0.95 (0.49–1.85)	1.02 (0.46–2.27)	1.50 (0.81–2.77)	1.48 (0.68–3.25)	**0.41 (0.17–0.97)** a	1.28 (0.76–2.17)	1.35 (0.72–2.54)
Problem drinking	N	1563	674	1639	1973	1447	1683	1610	2409	1685
	No	1.00	1.00	1.00	1.00	1.00	1.00	1.00	1.00	1.00
	Yes	1.16 (0.63–2.12)	1.30 (0.33–5.06)	1.48 (0.87–2.53)	1.85 (0.96–3.56)	1.55 (0.80–3.03)	**2.80 (1.61–4.86)^c^**	1.06 (0.69–1.62)	**1.72 (1.07–2.75)** a	1.48 (0.81–2.70)
Smoking	N	1605	1650	1677	1998	1694	1723	1667	2549	1768
	No	1.00	1.00	1.00	1.00	1.00	1.00	1.00	1.00	1.00
	Yes	1.02 (0.60–1.75)	1.03 (0.39–2.77)	0.99 (0.60–1.66)	1.34 (0.81–2.21)	1.32 (0.73–2.39)	**2.29 (1.36–3.86)^b^**	0.64 (0.40–1.03)	1.10 (0.72–1.69)	1.13 (0.68–1.87)
**Health outcome** ‡										
Poor self-rated health	N	1604	1650	1677	1994	1695	1723	1656	2540	1764
	No	1.00	1.00	1.00	1.00	1.00	1.00	1.00	1.00	1.00
	Yes	**2.25 (1.37–3.71)^b^**	**2.43 (1.27–4.65)^b^**	**3.02 (1.80–5.06)^c^**	**1.92 (1.29–2.86)^b^**	1.80 (0.93–3.48)	**2.34 (1.37–3.99)^b^**	0.89 (0.59–1.34)	**2.43 (1.60–3.69)^c^**	**1.77 (1.14–2.76)** a
Psychological distress	N	1515	1574	1571	1939	1626	1691	1559	2351	1620
	No	1.00	1.00	1.00	1.00	1.00	1.00	1.00	1.00	1.00
	Yes	**1.88 (1.21–2.92)^b^**	**3.42 (1.85–6.31)^c^**	**4.38 (2.79–6.88)^c^**	**3.58 (2.46–5.21)^c^**	**3.10 (1.84–5.25)^c^**	**2.63 (1.69–4.09)^c^**	**1.98 (1.39–2.81)^c^**	**2.61 (1.78–3.84)^c^**	**3.41 (2.22–5.25)^c^**

Adjusted for sex, age, marital status, education, location, household size, physical activity difficulty, locus of control, wealth, social support, and death of close relative.

# Loneliness was assessed by the question ‘How often do you feel lonely?’ and those who answered ‘Often’ were coded as 1 and ‘Sometimes/Rarely/Never’ as 0.

† Alcohol consumption was based on the question ‘How often do you consume alcoholic drinks of any type?’ with 8 main response categories ranging from ‘Every day’ to ‘Never’. Those who did not respond ‘Never’ were categorised as alcohol consumers. Heavy episodic drinking was usual consumption of at least one of the following on one occasion: ≥200 g of strong spirits (e.g. vodka), ≥2 litres of beer, ≥750 g of industrially produced wine/champagne. CAGE problem drinking was based on 4 questions: Have you ever felt you should cut down on your drinking?; Have people annoyed you by criticizing your drinking?; Have you ever felt bad or guilty about your drinking?; Have you ever had a drink first thing in the morning to steady your nerves or to get rid of a hangover (eye opener)? Answers to these questions were coded as 1 (yes) and 0 (no) and added to create a scale ranging from 0 to 4. Those who scored ≥2 on this scale were CAGE problem drinkers. Smoking referred to answering ‘Yes’ to the question ‘Do you smoke at least one cigarette (papirossi, pipe, cigar) per day?’.

‡ Poor self-rated health was claiming to have poor or very poor health in general. Psychological distress was assessed by a composite score based on 11 questions (refer to text for details) and referred to those with the highest quintile of scores.

a P<0.05, ^b^ P<0.01, ^c^ P<0.001.

## Discussion

To the best of our knowledge, this is the first study to examine the prevalence and correlates of loneliness, and its association with health behaviours and health outcomes across a number of countries in the FSU. It has shown that the prevalence of loneliness varies widely across the FSU countries and that there are no clearly discernible geographic patterns. Some of the correlates of feeling lonely were the same across all of the countries – while others were more country-specific. The effects of loneliness on health behaviours varied across countries. While lonely respondents in Kyrgyzstan were more likely to consume alcohol, engage in problem drinking and smoke, those in Russia drank in a problematic way. In Armenia feeling lonely was associated with a significantly increased risk of engaging in heavy episodic drinking, while in Moldova the risk for this form of alcohol consumption was reduced. Loneliness was associated with poor self-rated health in seven of the nine countries and with psychological distress in every country.

Recent research which focused on 25 countries throughout Europe (including Russia and Ukraine from the FSU) has suggested that rates of loneliness are higher in Eastern Europe and that there is an almost linear relationship between increasing age and feelings of frequent loneliness among the Eastern European countries [27]. By using a larger number of FSU countries, we showed that the prevalence of feeling lonely varies markedly among the countries in this region and that there is no discernible age pattern across the countries. It is not possible, with the available data, to ascertain the reasons why the prevalence of loneliness differs between the countries in this region although it might be explained, at least in part, by the differing prevalence of the correlates of feeling lonely. For example, the prevalence of loneliness was highest in Georgia and Moldova – countries where there were high levels of reported divorce/widowhood, personal bereavement and which have both experienced a high level of out-migration and temporary labour migration in recent years [37], [38], [39].

Several previous studies have hypothesised that ‘transition’ and its effects may be affecting levels of perceived loneliness in the countries in Eastern Europe [27], [40]. Recognising the difficulty in trying to operationalise this, in the current study, we examined the effects of a number of individual-level correlates that have been linked with feelings of loneliness more generally in other contexts, but the effects of which might have been more widespread during the period of transition as a result of the societal changes it has brought in its wake. In relation to this, we found that divorce and widowhood, living alone, having a low level of social support and experiencing the recent death of a close relative were associated with a higher likelihood of feeling lonely in either all or a majority of the countries. This is intuitive and accords with research from the West which has shown that processes which lead to loss and change in social relations underpin loneliness [2], [41].

Although findings have varied between studies, some previous research has indicated that feeling lonely may be linked to a number of risky health behaviours such as alcohol use and abuse [8], [42], [43], [44], smoking [6], [7], recreational drug use [45], and physical inactivity [46]. Several possible mechanisms have been proposed to explain why loneliness may lead to more hazardous health behaviour. It has been suggested for example, that social exclusion may be linked to poorer self-regulation and thus worse health behaviours [47]. It is also possible that behaviours such as smoking might be undertaken in an attempt to connect with others and gain social acceptance [6]. Loneliness has also been associated with reporting higher levels of stress [48], [49], and in such circumstances, behaviours such as consuming alcohol (and smoking) might be used in response [2], possibly in an attempt to mitigate its effects.

In the present study, we found a complex pattern as regards feeling lonely and risky health behaviours. In some countries there was no relationship; in several countries loneliness was associated with risky drinking, while in Moldova, loneliness was linked to a reduced risk of heavy episodic drinking. These varying results accord with the mixed findings from earlier studies [7], [45], [49], [50] and may indicate that country-specific antecedents, possibly relating to social or cultural factors, which some authors argue are important in terms of understanding between-country differences in loneliness more generally [51], [52], may also be important in terms of loneliness and risky health behaviours. In Kyrgyzstan for example, where loneliness was associated with smoking, alcohol consumption and problem drinking, recent research has highlighted how, in the face of a sharp economic decline, many people are now reliant on subsistence agriculture. In turn, this has been linked to comparatively high levels of population satisfaction, possibly because of the high levels of social contact and cooperation with known others that it entails [31]. In such an environment feeling lonely might be especially stressful and underpin the use of coping mechanisms such as alcohol and tobacco. Alternatively, in a society where there is a much greater tendency to follow the Islamic proscription on alcohol consumption than in neighbouring Kazakhstan [53], disapproved behaviours might themselves be a cause of ostracism, social isolation and loneliness. The relationship we observed between loneliness and problem drinking in Russia seems to accord with earlier research that has linked social marginalisation with alcohol use and mortality in the country [54], and suggests that isolation, whether objective or perceived, increases the risk of alcohol misuse in that context. However, the fact that loneliness was associated with a lower risk for heavy episodic drinking in Moldova was an unexpected finding. Overall, these complex results and their potentially important public health consequences clearly highlight the need for more detailed country-specific research on how loneliness is linked with different health behaviours in the countries in this region.

Feeling lonely was associated with poor self-rated health in seven countries and psychological distress in all of the countries. These results mirror those from a number of previous studies which have linked loneliness to both poor self-rated health [55], [56], and poorer mental health outcomes such as depression and anxiety [2], [57]. It is uncertain how loneliness might affect health although a number of possible pathways have been proposed ranging from more hazardous health behaviours and changes in physiological functioning through to a failure to take medications [40], [58]. Stress might also be a central element in this process. It has been argued that lonely individuals have greater exposure to stressors, perceive activities as being more stressful, use coping strategies that might perpetuate stress and exhibit an elevated physiological response to stress [30]. This possible connection with stress is important as stress has also been closely linked with depression [59], which might help explain the association we observed between feeling lonely and psychological distress in the current study.

There are several potential limitations to this study. First, we used a single-item question to measure loneliness. It has been argued that because there is great stigma attached to loneliness, direct questions which use the word lonely are likely to result in underreporting of the phenomenon [60] and that this may be especially pronounced among males [61]. It is possible therefore that our finding of significantly higher odds for loneliness among females in some countries might simply be an artefact of the measure used. Having said this, a number of authors have previously argued that single-item questions produce similar findings to multiple-item scales [62] and are generally robust when used with respondents at both ends of the distribution i.e. the not lonely or severely lonely categories [63]. Second, we used a frequency measure of loneliness and interpreted frequent loneliness as a more serious manifestation of this phenomenon. However, we were not able to examine how subjects perceived the intensity of this phenomenon – which might have been important in terms of health outcomes. Third, we cannot discount the possibility that some potentially important variables were not included in the analysis. Even though our focus was on the social correlates of loneliness, it would have been desirable to examine other factors, such as personality characteristics, which previous literature has suggested may be important when it comes to understanding loneliness [2], [61], but for which we had no data. Lastly, this study made use of cross-sectional data and could not therefore establish the temporal ordering of the associations observed. It is possible for example, that physical or mental ill health might be a precursor of loneliness rather than a result of it.

In conclusion, this study has shown that the prevalence of loneliness varies throughout the countries of the FSU, and that feeling lonely is associated with risky health behaviours in some countries and poorer health in every country. This suggests that loneliness might be an important, but until now, neglected public health problem in the countries in this region.
